# Supplementary figures and images for: Antiviral Activity of Acetylsalicylic Acid against Bunyamwera Virus in Cell Culture
Source: Viruses. 2023 Apr 11;15(4):948. doi: 10.3390/v15040948 (PMC10141918; doi:10.3390/v15040948)

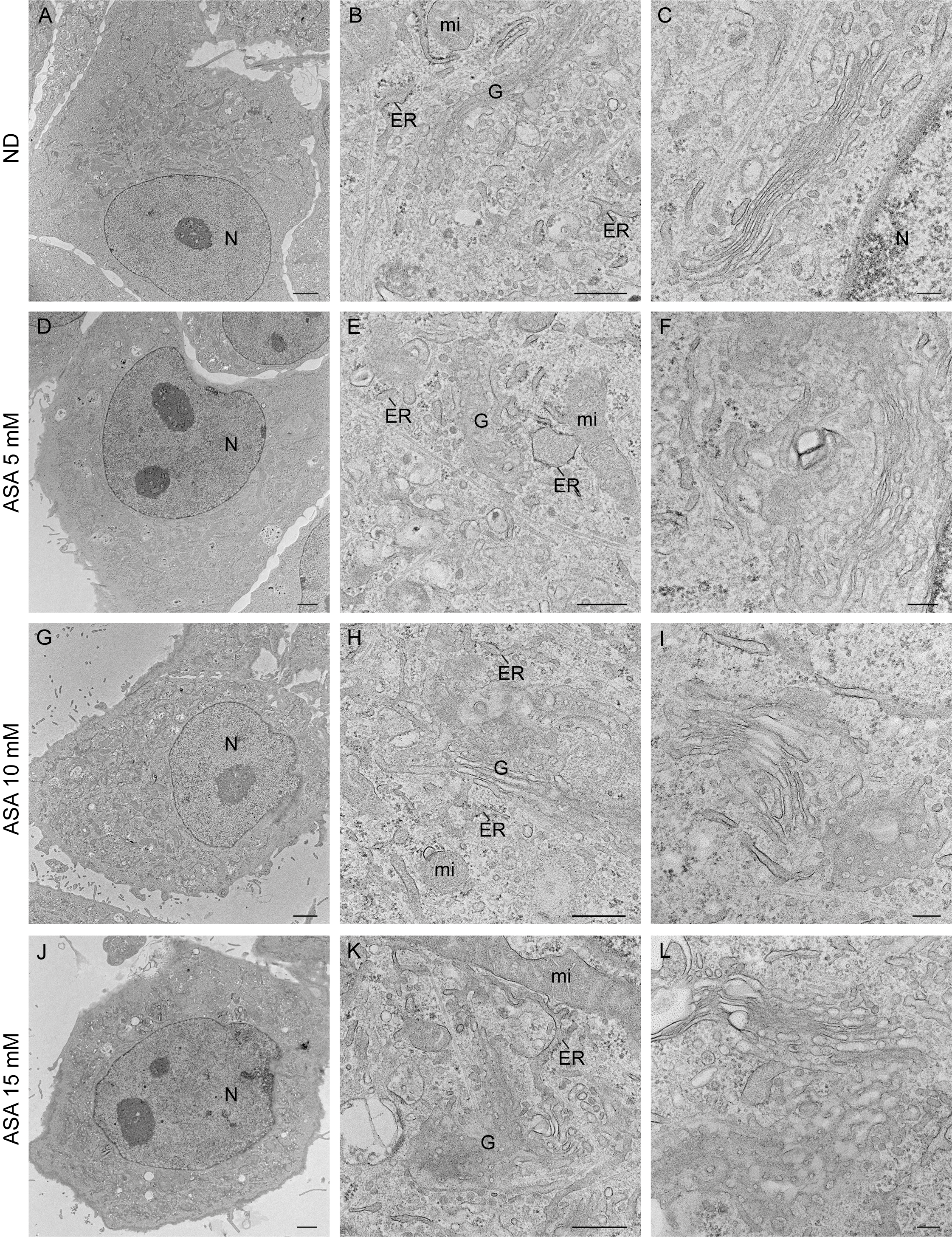

Supplement: Supplementary file 1 [file viruses-15-00948-s001.zip › Supplementary Figures/Figure S2.tif]

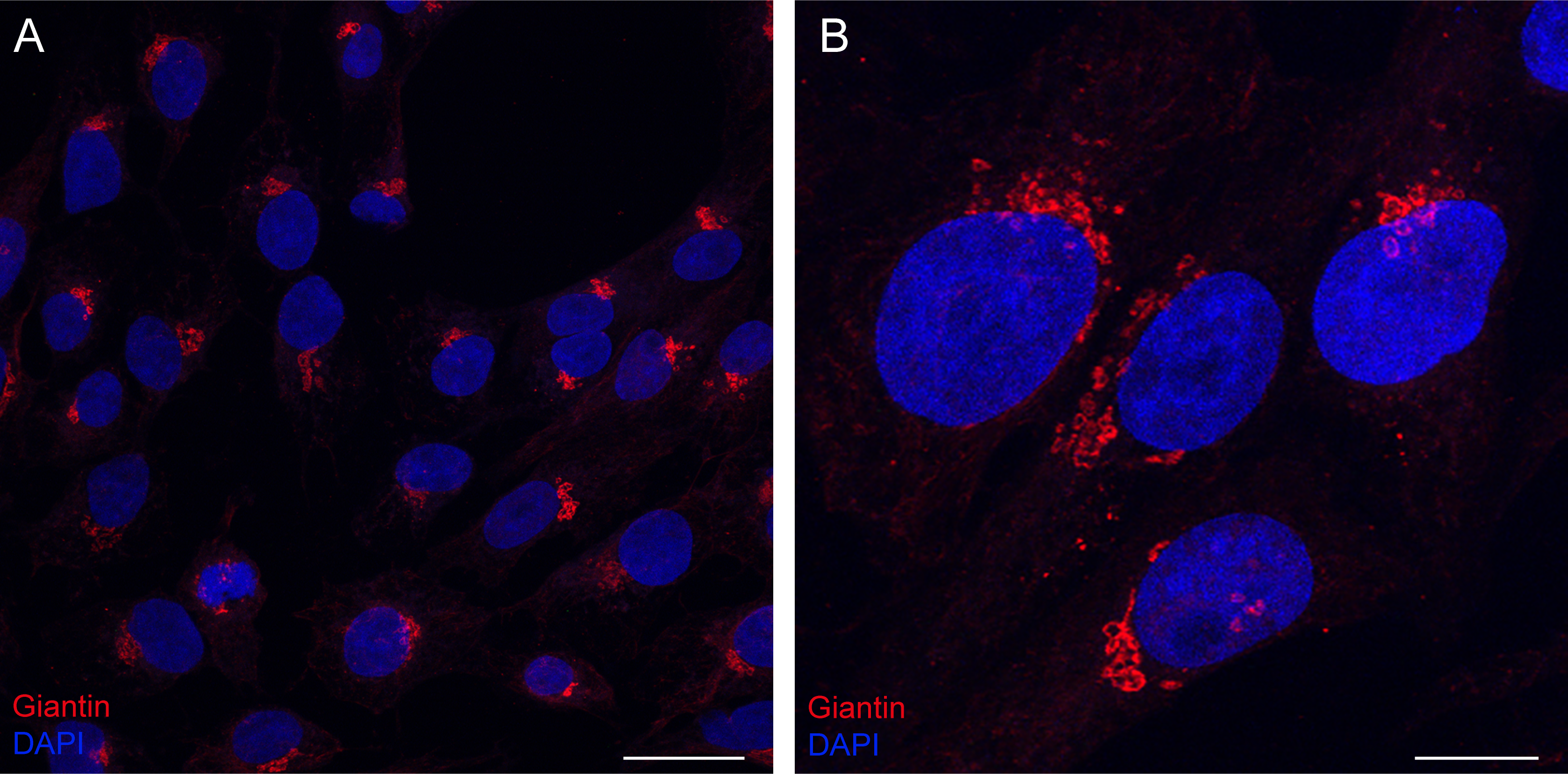

Supplement: Supplementary file 1 [file viruses-15-00948-s001.zip › Supplementary Figures/Figure S1.tif]
